# Glycemic Response in Nonhuman Primates Fed Gluten-Free Rice Cakes Enriched with Soy, Pea, or Rice Protein and Its Correlation with Nutrient Composition

**DOI:** 10.3390/nu16020234

**Published:** 2024-01-11

**Authors:** Yong Yang, Qingsu Liu, Feng Yue

**Affiliations:** 1 State Key Laboratory of Digital Medical Engineering, School of Biomedical Engineering, Hainan University, Sanya 572025, China; yangyong0507@163.com; 2 Collaborative Innovation Center of One Health, Hainan University, Haikou 570228, China; 3 Food, Water, Waste Research Group, Faculty of Engineering, The University of Nottingham, University Park Campus, Nottingham NG7 2RD, UK; qingsu.liu@nottingham.ac.uk

**Keywords:** gluten free, glycemic index, IAUC, nonhuman primates

## Abstract

Celiac disease (CD) is a chronic disease caused by the consumption of gluten foods and is closely related to type 1 diabetes (T1D). Adherence to a gluten-free (GF) diet is the cornerstone of treating CD, and certain plant proteins added to GF foods affect blood glucose to varying degrees. The aim of this study was to analyze and compare the changes in glycemic index (GI) and incremental area under the postprandial glucose tolerance curve (IAUC) of various foods through consumption of GF foods supplemented with certain plant proteins in non-human primates. The test foods were GF rice cakes with 5%, 10%, and 15% added single plant proteins (rice protein, soy protein, and pea protein) mixed with rice flour, as well as 5%, 10%, and 15% gluten rice cakes, and rice flour alone, for a total of 13 food items, and 12 healthy cynomolgus monkeys were examined for their glucose levels in the blood after fasting and after eating each test food (50 g) for 15, 30, 45, 60, 90, and 120 min after fasting and eating each test food. Fingertip blood glucose levels were measured, and the nutrient content of each food, including protein, fat, starch, ash, and amino acids, was examined. All foods tested had a low GI (<50) when analyzed using one-way ANOVA and nonparametric tests. Postprandial IAUC was significantly lower (*p* < 0.05) for GF rice cakes with 15% pea protein (499.81 ± 34.46) compared to GF rice cakes with 5% pea protein (542.19 ± 38.78), 15% soy protein (572.94 ± 72.74), and 15% rice protein (530.50 ± 14.65), and GF rice cakes with 15% wheat bran protein (533.19 ± 34.89). A multiple regression analysis showed that glycine was negatively associated with IAUC in GF rice cakes with 5%, 10%, and 15% pea protein added (*p* = 0.0031 < 0.01). Fat was negatively correlated with IAUC in GF rice cakes supplemented with 5%, 10%, and 15% soy protein (*p* = 0.0024 < 0.01). In this study, GF rice cakes made with added pea protein were superior to other gluten and GF rice cakes and had a small effect on postprandial glucose.

## 1. Introduction

Rapid changes in the lifestyles of people around the world have occurred due to economic globalization, urbanization, and fast-paced development in recent years [1]. These changes have significantly impacted people’s dietary habits, leading to poor health among many people due to the foods they consume. Gluten, a complex protein found in many grains [2], contains alcohol-soluble proteins that are known to be pathogenic factors for celiac disease (CD) [3] and have been implicated in other diseases such as type 1 diabetes [4], rheumatoid arthritis [5], and multiple sclerosis [6], among others [7,8,9]. Increasing evidence indicates that a considerable number of patients exhibit neurological dysfunction. The first systematic evidence of gluten-related neurological disease dates back to 1966 when electron microscopy and hematoxylin and eosin staining were used to examine neurodegenerative changes in muscle biopsies of adult patients with CD. Subsequent studies reported the first fatal case caused by neurological disease, comparing autopsy results with nine other CD cases presenting progressive central nervous system disorders. The case showed a common histopathological feature of progressive neurodegeneration in the cerebellum, deep gray matter, brainstem, and spinal cord [10]. Another study demonstrated that following a gluten-free (GF) diet stabilized not only gastrointestinal symptoms but also neurological symptoms in patients with CD [11]. This demonstrated that the long-term consumption of gluten led to neurological dysfunction. Moreover, GF food is the only way to achieve complete symptom relief among patients with CD [12].

Diabetes mellitus (DM) is a chronic, systemic metabolic disease characterized by elevated blood glucose levels [13]. Type 1 DM (T1DM) has a high comorbidity with CD [7,14]. Long-term gluten consumption also increases the risk of developing T1DM [15]. Early studies in mice suggest a role of gluten in the pathogenesis of T1DM, as it alters the composition of the gut microbiota, which may further promote the development of T1DM [16]. A lifelong GF diet reduced the incidence of autoimmune diabetes from 64% to 15% [17]. Therefore, dietary changes are crucial for achieving and maintaining metabolic control and help reduce the burden of diabetes-related complications [18].

GF foods refer to those that are completely free of gluten or do not contain gluten-containing grains [19]. Although significant progress has been made in understanding and improving GF foods over the past two decades through assessing different ingredients, additives, and technologies, finely processed GF foods may contain higher amounts of sugar and oil, increasing the risk of diabetes and obesity. The development of GF products still faces challenges. Currently, the focus is more on plant-based functional foods because their health benefits primarily come from plant proteins. Some plant proteins are typically added to GF foods to improve their structure, texture, and glycemic control [19]. Developing plant-protein-based GF foods is an important trend, especially in managing CD and diabetes. Rice is the second largest consumed cereal after wheat. It is a highly nutritious food source, rich in protein and an excellent source of carbohydrates, besides containing various vitamins and minerals such as niacin and thiamine [20]. Also, it is GF, making it a good choice for individuals with gluten sensitivity. Rice flour also plays a vital role as an ingredient in traditional and new food products [21,22,23], and frequently is the main ingredient applied in GF products both from food industry and food scientists. However, rice has a glycemic index (GI) ranging from 54 to 121 [24]. The long-term consumption of foods with a GI between 79 and 82 significantly increases the risk of developing diabetes [25]. Consuming certain different types of plant-based proteins can reduce the incremental area under the blood glucose curve (iAUC) after a meal [26], which can help control blood sugar levels. Therefore, rice is not the most appropriate food choice for patients with diabetes. Legumes such as soy and peas are an important source of plant proteins and are rich in carbohydrates, fibers, proteins, and various micronutrients [27]. Consuming legume protein is associated with a reduced incidence of diabetes [28]. Therefore, it is recommended that individuals choose low-GI GF foods rich in legume protein, which helps control blood sugar levels and alleviates related diseases.

In the development of GF foods, GI is a way to evaluate the speed and extent of the rise in blood sugar caused by the carbohydrates present in food within 2 h of consumption [29]. Compared with regular foods, most GF foods have a lower GI and can help control blood sugar [30]. The incremental area under the glucose curve (IAUC), calculated by measuring the increase in the blood sugar level after a meal relative to the fasting level, serves as an evaluation index for the blood glucose response to each test meal. GI refers to the percentage increase in the area under the blood glucose curve after consuming a target amount (usually 50 g) of available carbohydrates in a test food compared with the corresponding increase after consuming the same amount of available carbohydrates in a reference food (such as glucose). Foods are classified into high GI (>70), medium GI (55–70), and low GI (<55) based on their GI values. High-GI foods can lead to a rapid release of carbohydrates, resulting in an increase in blood glucose concentration [31]. In contrast, low-GI foods have a slower digestion and absorption rate, leading to a gradual rise in blood glucose levels [32]. This positively affects the prevention and management of diabetes, obesity, cardiovascular disease, hyperlipidemia, and hypercholesterolemia [33,34]. For patients with both CD and T1DM, maintaining good glycemic control and adhering to a strict low-GI GF diet is essential to avoid complications associated with these two diseases.

Nonhuman primate (NHP) models have been successfully established for studying CD-related issues [35]. By feeding NHPs with gluten-containing food, gluten-sensitive macaques exhibited obvious CD symptoms, including chronic diarrhea, fat malabsorption, and intestinal lesions. These clinical, histological, and serological features were reversed through intervention with a GF diet [36]. Therefore, these NHP models are of great significance for studying both fundamental and practical CD-related issues [37]. Moreover, NHP models have been used to investigate the development and pathophysiological changes of obesity and diabetes [38]. The blood glucose regulation and pathological characteristics exhibited by the cynomolgus monkeys are quite similar to the clinical features of certain human diseases. Additionally, captive macaques have a longer lifespan, and their living environment and dietary habits are relatively fixed and uniform, making it more feasible to study diseases through dietary interventions in NHPs [39]. Thus, NHP models can effectively help with learning about many diseases and facilitate long-term research on the effects of dietary interventions on animals.

This study aimed to investigate the effect of GF rice cakes made with different ratios of plant-based protein and rice flour, and whole-grain rice cakes, on the postprandial blood glucose response in nonhuman primates. Thirteen test foods were prepared, including GF rice cakes mixed with 5%, 10%, or 15% of a single plant-based protein (rice protein, soy protein, and pea protein) and whole-grain rice cakes mixed with 5%, 10%, or 15% wheat protein as well as plain rice flour. Twelve healthy cynomolgus macaques were tested for fasting and postprandial fingertip blood glucose levels 15, 30, 45, 60, 90, and 120 min after consuming each test food (50 g). The GI and IAUC were analyzed for each test food, and the nutritional components, including protein, fat, starch, ash, and amino acids, were determined. It provides valuable insights into the selection and long-term consumption of GF foods with added plant-based protein for blood glucose control. This is particularly important for those with gluten intolerance and diabetes, and those who regularly consume gluten-containing foods and are at risk of developing neurodegenerative diseases.

## 2. Materials and Methods

### 2.1. Experimental Animals

Before starting the experiment, all animals were screened for health and had normal blood and biochemical parameters that met the experimental criteria. Table 1 summarizes the physical signs of 12 healthy male cynomolgus monkeys aged 12–16 years. These 12 cynomolgus monkeys were naïve and had never been involved in any pharmacological tests or studies before the experiment. During the study period, the animals were kept in stainless steel monkey cages in the nonhuman primate facility of Thinxon Biomedical Co., Ltd. (Nanning, China), which obtained a laboratory animal use license accredited by Guangxi province. The animals were fed normally during the experimental period without trial feeding, twice a day, and supplemented with fresh fruit once a day. All animals were kept at room temperature of 22–28 °C for 12 h with light and dark cycles (7:00 a.m. to 7:00 p.m.). The relative humidity was 30–75%, and water was consumed ad libitum. All experiments were performed during the daytime. In addition, animals underwent a thorough examination before the experiments and were confirmed to be free of other diseases such as tuberculosis. The study protocol was approved by the Institutional Animal Care and Use Committee (IACUC). The approval number is SSLl-21002, and the approval time is 22 August 2021.

The animals were screened as follows: (1) body mass index (BMI) < 35 kg/m^2^; (2) body weight and other biochemical parameters such as fasting glucose within normal limits; (3) no history of a neurological or psychiatric diagnosis; (4) no history of drug/alcohol abuse; (5) not currently taking any psychoactive drugs, nutrient supplements affecting glucose tolerance within 3 months of participation in any other study, and oral contraceptives, acetylsalicylic acid, steroids, protease inhibitors, and so forth; (6) screening of monkeys not infected with chronic diarrhea through fecal testing.

### 2.2. Experimental Material

The materials required for the experiment are presented in Table 2.

### 2.3. Experimental Methods

#### 2.3.1. Test Food Preparation Method

SPI, PPI, RPI, and WPI were mixed into rice flour (where PI stands for single protein and is replaced sequentially according to the corresponding protein) in proportion to each other (meal formulation as in Table 3), and the batter was formed by pouring water into the powdered mixture. The moisture content of the raw batter reached 65%. The batter (about 25 g) was transferred to aluminum molds and steamed for 20 min. The rice cakes were then demolded, cooled at room temperature for 5 min, and stored in an airtight container. The samples were freeze-dried, and the contents of proteins, amino acids, and so forth were determined. A certain amount of sugar-free sweetener (not more than 0.5 g/100 g in solids) was added appropriately according to the national food addition standards, considering the taste. For LAGG, the dosage was 1%. That is, 0.35 g of LAGG was added to 35 g of rice flour.

#### 2.3.2. Reference Food Preparation Method

An appropriate amount of food-grade anhydrous dextrose (50 g) was dissolved in 250 mL of purified water. After nasal gavage within 2 min, blood glucose values were measured using a glucometer with a pinprick of fingertip blood at 0, 15, 30, 45, 60, 90, and 120 min.

### 2.4. Feeding Cycle

Taking WPI as an example, the food-feeding cycles are presented in Table 4. Both food feeding and data collection were conducted on days 1, 5, 9, and 13 of the experiment. Day 0 animals were fasted for ≥16 h. Twelve healthy experimental animals were kept on the same diet for four feeding cycles, and the animals in each cycle were fed the rice cake with 15%, 10%, and 5% PI, with the following feeding cycles: (1) Thai fragrant rice + wheat protein; (2) Thai fragrant rice + soy protein; (3) Thai fragrant rice + pea protein; and (4) Thai fragrant rice + rice protein. PI protein was replaced with SPI, PPI, and RPI sequentially according to the experimental advancement.

### 2.5. Experimental Operation Method

The experimental animals maintained regular rest and a normal diet. High-dietary-fiber and high-sugar foods were avoided at dinner the day before the measurement, fasting (≥16 h) was started, and strenuous exercise was avoided in the early morning of the measurement day. Two weeks before the start of the experiment, all experimental animals were acclimatized in a monkey chair to avoid severe stress reactions in the experimental process. The experimental animals were rested in the monkey chair for 10 min after 5 min. The glucose levels were recorded twice from the fingertips of the animals under the fasting condition. Then, the animals were allowed to start eating, and the eating time was strictly controlled, starting from the first bite of eating time. The eating was completed within 5–10 min. The blood was collected 15, 30, 45, 60, 90, and 120 min after the meal and measured using the glucose meter; if necessary, the blood collection time could be extended (e.g., 180 min). The consistency and accuracy of blood collection time points were ensured. Consistent blood collection sites should be used during the measurement cycle, and feces should be collected on the day of the experiment and frozen at −80 °C.

### 2.6. Statistical Analysis

The data were statistically analyzed and plotted using GraphPad Prism 9.0.0 (GraphPad Software, Inc., San Diego, CA, USA), IBM SPSS Statistics 27.0.1 (SPSS, Inc., Chicago, IL, USA), and Origin 2021b (Origin Lab Corporation, Northampton, MA, USA) software. Data are presented as the mean ± standard error (S) unless otherwise noted. The quantitative data were presented as the mean ± standard error. The within-group and between-group comparisons were analyzed using independent-sample *t* tests and nonparametric tests. For all data analyses, * *p* < 0.05 and ** *p* < 0.01 were considered to indicate statistical significance. BMI was calculated as follows: BMI = weight/height^2^ (where height is the length from the crown of the head to the buttocks).

## 3. Results

### 3.1. Postprandial Glycemic Response and GI of Reference and Test Foods Consumed by Healthy Experimental Animals

The foods were tested once, and the glucose reference beverages (50 g of glucose) were each tested twice.

**Figure 1:**
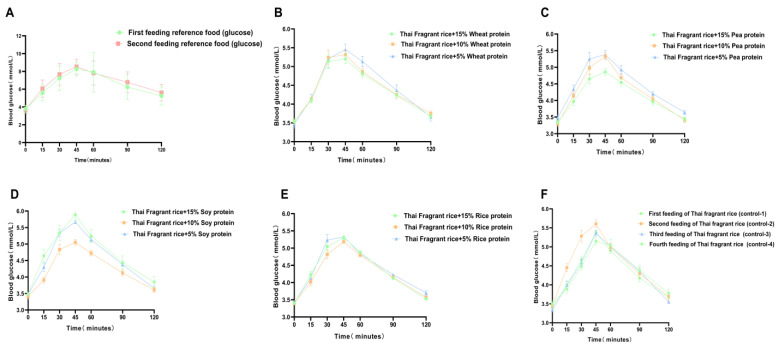
Changes in mean blood glucose concentrations of healthy cynomolgus monkeys (*n* = 12) after ingesting the reference food and the test food (mean ± SD). Note: (**A**) Plots of mean blood glucose concentration changes in healthy cynomolgus monkeys (*n* = 12) after two infusions of 500 g/L glucose solution (reference food) (mean ± SD). (**B**) Plots of mean blood glucose concentration changes in healthy cynomolgus monkeys (*n* = 12) after consuming gluten rice cakes with three proportions (5%, 10%, and 15%) of wheat protein (mean ± SD). (**C**) Plots of mean blood glucose concentration changes in healthy cynomolgus monkeys (*n* = 12) after consuming GF rice cakes with three proportions (5%, 10%, and 15%) of soy protein (mean ± SD). (**D**) Plots of mean blood glucose concentration changes in healthy cynomolgus monkeys (*n* = 12) after consuming GF rice cakes with three proportions (5%, 10%, and 15%) of soy protein (mean ± SD). (**E**) Plots of mean blood glucose concentration changes in healthy cynomolgus monkeys (*n* = 12) after consuming three proportions (5%, 10%, and 15%) of rice protein (mean ± SD). (**F**) Mean blood glucose concentration changes in healthy cynomolgus monkeys (*n* = 12) after four servings of GF rice cakes (control) with pure rice flour (mean ± SD).

**Figure 2:**
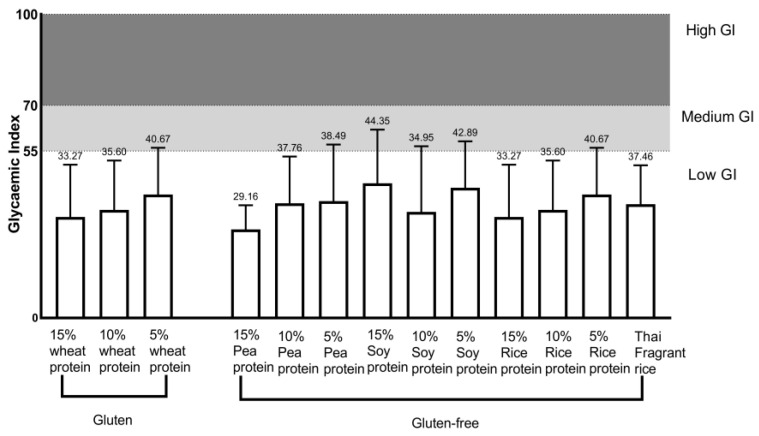
Glycemic index (GI) of 13 gluten-containing and GF tested foods (mean ± SE).

**Figure 3:**
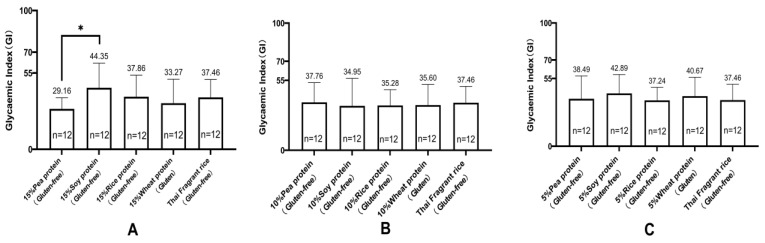
Glycemic index (GI) of gluten-containing and gluten-free rice cakes with the same proportion of vegetable protein (mean ± SE). Note: (**A**) Glycemic index (GI) of rice cakes with 15% wheat protein and 15% gluten-free vegetable protein added and rice cakes with plain rice flour (mean ± SEM). (**B**) Glycemic index (GI) of rice cakes supplemented with 10% wheat protein and 10% gluten-free vegetable protein and plain rice flour rice cakes (mean ± SEM). (**C**) Glycemic index (GI) of rice cakes with 5% wheat protein and 5% gluten-free vegetable protein and plain rice flour rice cakes (mean ± SEM). * *p* < 0.05.

**Figure 4:**
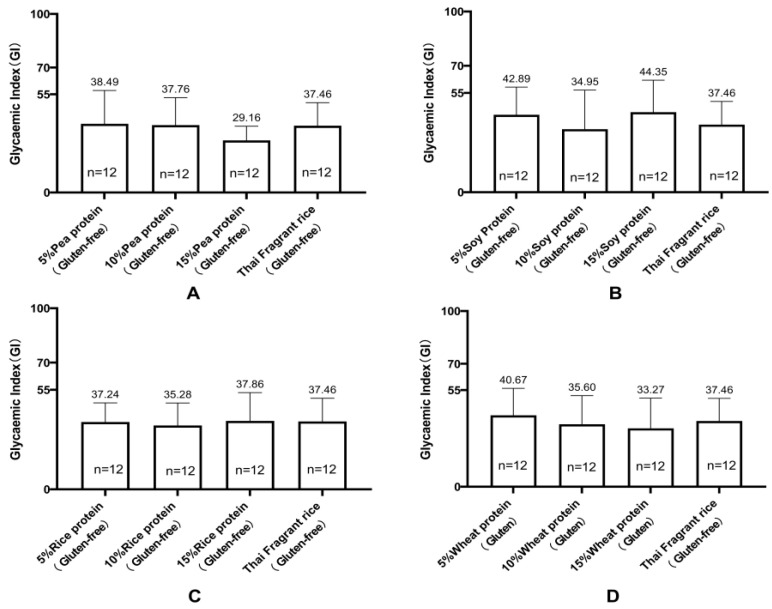
Glycemic index (GI) of rice cakes with and without gluten protein added to the same plant-based protein (mean ± SE). Note: (**A**) Glycemic index (GI) of rice cakes with 5%, 10%, and 15% pea protein and rice cakes with pure rice flour (mean ± SE). (**B**) Glycemic index (GI) of rice cakes with 5%, 10%, and 15% soy protein and plain rice flour (mean ± SE). (**C**) Glycemic index (GI) of rice cakes with 5%, 10%, and 15% rice protein and pure rice flour (mean ± SE). (**D**) Glycemic index (GI) of rice cakes with 5%, 10%, and 15% wheat protein and pure rice flour (mean ± SE).

Figure 1A shows the changes in the mean blood glucose levels in healthy cynomolgus monkeys (*n* = 12) following two infusions of a 200 g/L glucose solution. Figure 1B–F indicate the change in mean postprandial blood glucose concentration in healthy cynomolgus monkeys (*n* = 12) after ingestion of the test food, which showed a peak at 45 min and a near return to baseline within 2 h (Figure 1). Among all gluten-containing and GF rice cakes made with different concentrations of added vegetable protein and rice flour, GI values ranged from 29 to 47 (Figure 2 and Table 5). They were all at the low-GI (low GI < 50) level. GF rice cakes made from 15% pea protein and rice flour had a lower GI value (29.16 ± 7.96), and GF rice cakes made from 15% soy protein and rice flour had a lower high GI value (44.35 ± 17.68). The GI of rice cakes with gluten-containing and GF proteins under the condition of adding the same proportion of vegetable protein is illustrated in Figure 3. The GI of rice cakes with gluten-containing and GF protein under the condition of adding the same vegetable protein is depicted in Figure 4. In rice cakes with 15% wheat protein and 15% GF vegetable protein (Figure 3A), the GI of GF rice cakes with 15% pea protein was significantly lower than the GI of the GF rice cake with 15% soy protein (*p* < 0.05).

### 3.2. IAUC 0–120 min after the Intake of 13 Gluten-Containing and GF Tested Foods in Healthy Experimental Animals

**Figure 5:**
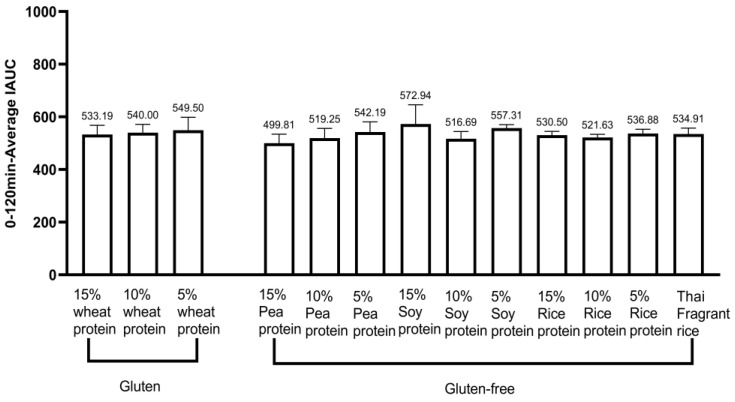
Incremental area under the 0–120 min postprandial glucose curve (IAUC) for 13 gluten-containing and gluten-free tested foods (mean ± SE).

**Figure 6:**
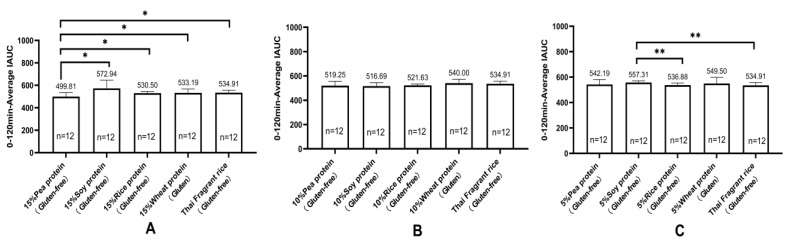
Incremental area under the postprandial blood glucose curve (IAUC) for gluten and rice cakes without gluten protein under the condition of adding the same proportion of vegetable protein (mean ± SE). Note: (**A**) Incremental area under the postprandial 0–120 min glucose curve (IAUC) for rice cakes and pure rice flour rice cakes supplemented with 15% wheat protein and 15% gluten-free vegetable protein (mean ± SE). (**B**) Incremental area under the postprandial 0–120 min glucose curve (IAUC) for rice cakes and pure rice flour rice cakes supplemented with 10% wheat protein and 10% gluten-free vegetable protein (mean ± SE). (**C**) Incremental area under the postprandial 0–120 min glucose curve (IAUC) for rice cakes supplemented with 5% wheat protein and 5% gluten-free vegetable protein and pure rice flour rice cakes (mean ± SE). * *p* < 0.05; ** *p* < 0.01.

**Figure 7:**
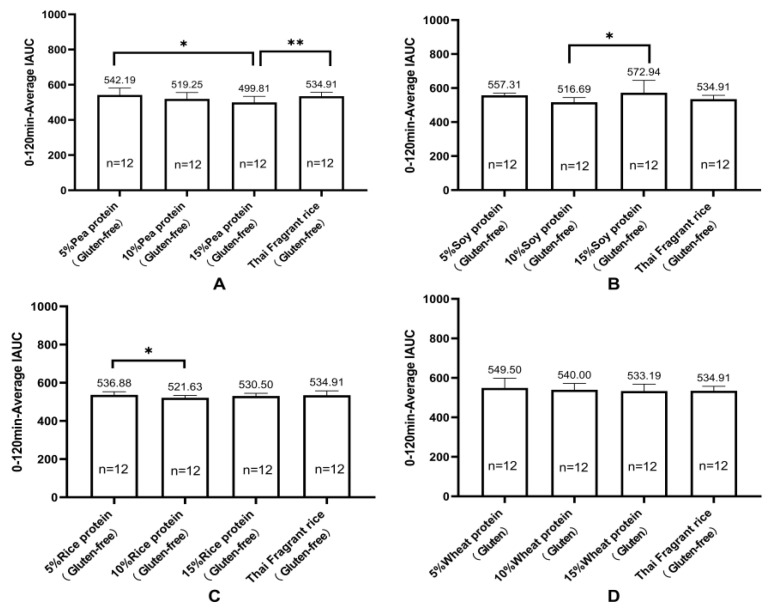
Incremental area under the postprandial 0–120 min glucose curve (IAUC) for gluten-containing and gluten-free protein rice cakes under the condition of adding the same plant-based protein (mean ± SE). Note: (**A**) Incremental area under the postprandial 0–120 min blood glucose curve (IAUC) for rice cakes with 5%, 10%, and 15% pea protein and pure rice flour added (mean ± SE). (**B**) Incremental area under the postprandial 0–120 min blood glucose curve (IAUC) for rice cakes with 5%, 10%, and 15% soy protein and pure rice flour added (mean ± SE). (**C**) Incremental area under the postprandial 0–120 min blood glucose curve (IAUC) for rice cakes with 5%, 10%, and 15% rice protein and pure rice flour rice cakes added (mean ± SE). (**D**) Incremental area under the postprandial 0–120 min blood glucose curve (IAUC) for rice cakes with 5%, 10%, and 15% wheat protein and pure rice flour rice cakes added (mean ± SE). * *p* < 0.05; ** *p* < 0.01.

Each of the healthy experimental animals consumed the test food, and their blood glucose levels were measured at various intervals: before eating and 15, 30, 45, 60, 90, and 120 min after eating. IAUC was calculated using the trapezoidal rule for the 0 to 120 min period to determine the differences in blood glucose kinetics. Table 6 and Figure 5 illustrate the IAUC for healthy cynomolgus monkeys (*n* = 12) who consumed gluten-containing and GF versions of the 13 tested foods. Figure 6 shows the 0 to 120 min postprandial IAUC for gluten-containing and GF rice cakes with the same proportion of added vegetable protein. The 0 to 120 min postprandial IAUC for GF rice cakes with 15% pea protein (499.81 ± 34.46) was significantly lower than that for GF rice cakes with 15% soy protein (572.94 ± 72.74), and 15% rice protein (530.50 ± 14.65), and 15% wheat protein in gluten rice cakes (533.19 ± 34.89) (*p* < 0.05). Furthermore, the 0 to 120 min postprandial IAUC for GF rice cakes made with 15% pea protein (499.81 ± 34.46) was significantly lower than that for GF rice cakes made with pure rice flour (534.91 ± 22.51) (*p* < 0.01). Finally, the 0 to 120 min postprandial IAUC for GF rice cakes with 5% soy protein (542.19 ± 38.78) was significantly higher than that for both GF rice cakes with 5% rice protein (536.88 ± 16.07) and the one with pure rice flour (534.91 ± 22.51). The 0 to 120 min postprandial IAUC for GF rice cakes with 15% soy protein (572.94 ± 72.74) was significantly higher than that for GF rice cakes with 10% soy protein (516.69 ± 27.82) (*p* < 0.01). Figure 7 illustrates the 0 to 120 min postprandial IAUC of rice cakes with and without gluten protein under the condition of adding the same vegetable protein. The study found that the 0 to 120 min postprandial IAUC of GF rice cakes with 15% pea protein (499.81 ± 34.46) was significantly lower than that of GF rice cakes with 5% pea protein (542.19 ± 38.78) (*p* < 0.05). Additionally, the 0 to 120 min postprandial IAUC for GF rice cakes with 15% pea protein (499.81 ± 34.46) was significantly lower than that for GF rice cakes made from pure rice flour (534.91 ± 22.51) (*p* < 0.01). Moreover, the 0 to 120 min postprandial IAUC for GF rice cakes with 15% soy protein (572.94 ± 72.74) was significantly higher than that for GF rice cakes with 10% soy protein (516.69 ± 27.82) (*p* < 0.01). The 0 to 120 min postprandial IAUC for GF rice cakes with 10% rice protein (521.63 ± 12.06) was significantly lower than that for GF rice cakes with 5% rice protein (536.88 ± 16.07) (*p* < 0.05).

### 3.3. Nutritional Composition of 13 Gluten-Containing and GF Tested Foods

**Figure 8:**
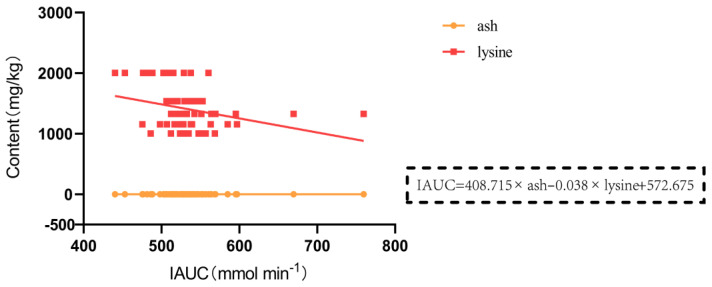
Relationship between the incremental area under the glycemic curve (IAUC) and the contents of various nutrients in gluten-containing and gluten-free protein rice cakes and pure rice flour rice cakes at 0 to 120 min postprandial under the condition of 15% added vegetable protein. Note: IAUC, and the content of lysine and ash.

**Figure 9:**
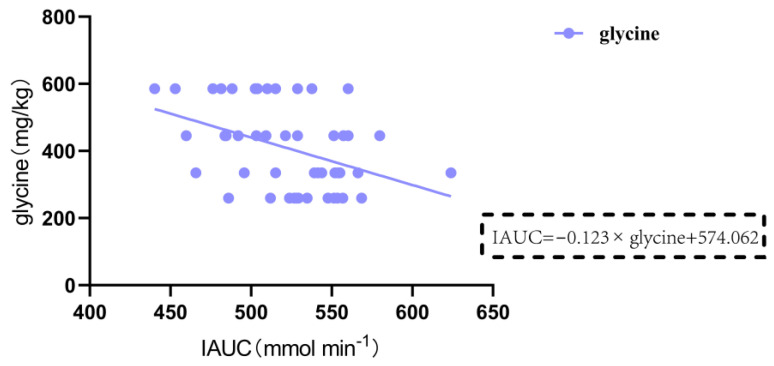
Relationship between the incremental area under the postprandial blood glucose curve (IAUC) and the content of various nutrients for 0–120 min with the addition of 5%, 10%, and 15% pea protein and pure rice flour rice cakes. Note: IAUC and the content of glycine.

**Figure 10:**
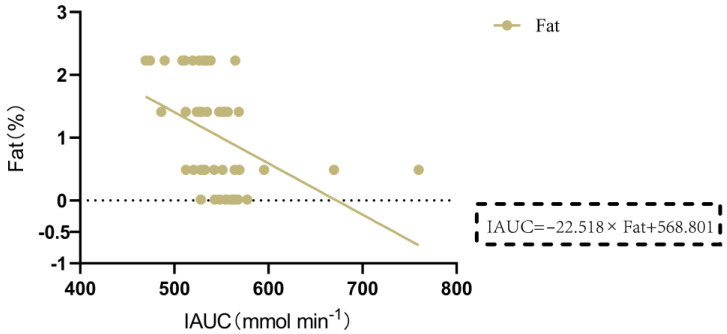
Relationship between the incremental area under the postprandial glucose curve (IAUC) and the contents of various nutrients for 0–120 min of rice cakes with 5%, 10%, and 15% soy protein and pure rice flour. Note: IAUC and fat content.

The nutritional composition of food is one of the factors affecting the postprandial glycemic response. Therefore, we analyzed the nutritional composition of 13 gluten-containing and GF tested foods, including protein, fat, starch, ash, and amino acids. The nutritional properties and composition of the different food types are presented in Table 7 and Table 8. We used the statistical method of a multiple regression analysis to investigate the relationship between the IAUC of blood glucose from 0 to 120 min after a meal and various types of nutritional components. The results showed that the content of lysine was negatively correlated with IAUC (*p* = 0.013 < 0.05) whereas the content of ash was positively correlated with IAUC (Figure 8) (*p* = 0.001 < 0.01) in gluten-containing rice cakes and those made from pure rice flour without gluten, which had 15% added plant-based protein. The regression equation was IAUC = 408.715 ash − 0.038 lysine + 572.675, with an *F*-value of 9.754 and a *p* value of <0.01, indicating that the fitted equation had statistical significance. The *R*^2^ value was 0.255, indicating a less-than-ideal fit, but the Variance Inflation Factor (VIF) value was 1.001 < 5, indicating no collinearity between variables. Additionally, the Durbin–Watson test value was 2.278, showing that the observations in the model were independent of each other. A highly significant negative correlation was found between glycine and IAUC in GF rice cakes and pure rice flour GF rice cakes with 5%, 10%, and 15% added pea protein (Figure 9) (*p* = 0.003 < 0.01). The regression equation was IAUC = −0.123 × glycine + 574.062, with an *F*-value of 9.719 and a *p* value of <0.01, indicating that the fitted equation had statistical significance. The *R*^2^ value was 0.174, indicating a less-than-ideal fit, but the VIF value was 1.000 < 5, indicating no collinearity between variables. Additionally, the Durbin–Watson test value was 2.559, showing that the observations in this model were independent of each other. A highly significant negative correlation was observed between fat content and IAUC in GF rice cakes and pure rice flour GF rice cakes with 5%, 10%, and 15% added soy protein (Figure 10) (*p* = 0.002 < 0.01). The regression equation was IAUC = −22.518 × fat + 568.801, with an *F*-value of 10.359 and a *p* value of <0.01, indicating that the fitted equation had statistical significance. The *R*^2^ value was 0.184, indicating a less-than-ideal fit, but the VIF value was 1.000 < 5, indicating no collinearity between variables. Additionally, the Durbin–Watson test value was 2.291, showing that the observations in this model were independent of each other.

## 4. Discussion

A high incidence of CD and T1DM [40] is considered a risk factor for metabolic diseases. Therefore, a critical task for these patients is to maintain good blood sugar control while adhering to a strict GF diet. Postprandial hyperglycemia is a significant concern for those diagnosed with prediabetes or diabetes, as it can lead to elevated glycated hemoglobin; such patients are advised to consume low-GI foods. The main determinants of postprandial glycemic response are the amount of carbohydrates ingested and the common constituents of the entire food (such as water, fat, protein, and fiber), processing techniques, and external factors. Some amino acids also affect postprandial glycemic response. Currently, only a few studies have examined the impact of GF diets on patients with T1DM and CD [41]. Also, only a limited number of small prospective and retrospective studies have discussed the glycemic benefits of GF diets [42]. Previous research has demonstrated a significant difference in glucose response to GF pasta between healthy participants and patients with CD, with healthy individuals exhibiting a significantly higher glucose response than patients with CD [43]. A study found that the fasting blood glucose levels increased significantly after 12 months on a GF diet [44]. In healthy individuals, the postprandial blood glucose response was higher with GF bread than with bread containing gluten [43]. Therefore, some GF foods may not be suitable for patients with abnormal glucose metabolism, and hence, further research on GF foods is needed. Although GF products are essential dietary needs for patients with CD, consumers without celiac disease need to consider that GF products are not necessarily a “healthier” food choice. Consumers should choose GF products that meet their individual needs while also paying attention to a balanced diet to avoid the negative effects of certain foods. The introduction of the GI concept provides a basis for patients with diabetes to make rational choices about carbohydrate-containing foods. A low-GI diet improves insulin sensitivity, lowers plasma triglyceride levels, reduces the risk of diabetes and heart disease, and helps treat obesity [45]. Low-GI foods stay in the digestive tract longer, have a lower absorption rate, and release glucose slowly, leading to lower postprandial blood glucose responses. The changes in postprandial blood glucose responses represent the balance between glucose entering and exiting the bloodstream. Lowering the GI of foods is a promising method, especially by adding some plant-based protein substitutes for traditional refined flour and starch materials, such as non-gluten grains and legumes, instead of using refined basic flours and starches, such as rice and corn flour, corn, potatoes, and cassava starch, because increasing the protein content can alter the digestion rate of starch, dilute the amount of available carbohydrates, and lower the GI.

Research has shown a significant correlation between the consumption of legume protein and a reduced incidence of diabetes. Legume protein can mitigate postprandial blood glucose response. Green pea legumes produce lower blood glucose peak responses in mixed diets with different types of legumes. Chickpeas, lentils, and green peas generate significantly lower postprandial blood glucose responses than pasta (~35%). Isolated green pea protein produces a lower glucose area under the curve (AUC), making it a valuable food component for improving blood sugar control [46], which is consistent with our research results. Some plant-based proteins are widely used to produce GF products. In this study, pea protein, rice protein, soy protein, and wheat protein were selected and processed into gluten-containing and GF rice cakes to examine their impact on postprandial blood glucose response in nonhuman primates. The GI values of the 10 GF rice cakes and 3 gluten-containing rice cakes ranged from 29 to 47, all with low GI (GI < 50). Rice cakes made from 15% pea protein and rice flour had a lower GI, while those made from 15% soy protein and rice flour had a higher GI. Meanwhile, the IAUC of postprandial blood glucose was the lowest for GF rice cakes made from 15% pea protein and rice flour. The IAUC of postprandial blood glucose was also highest for GF rice cakes made from 15% soy protein and rice flour. Additionally, the IAUC of GF rice cakes made with 15% pea protein was significantly lower than that of GF rice cakes made with 5% pea protein, 15% soy protein, 15% rice protein, and 15% wheat protein to produce gluten-containing rice cakes. Adding pea protein to make GF rice cakes had a minimal impact on postprandial blood glucose, which has important implications for a deeper understanding of the impact of nutrient content on blood sugar and the development of healthier food products. The impact of amino acids on blood glucose levels is mainly related to their metabolic pathways. Specifically, when branched-chain amino acids, including leucine, isoleucine, and valine, and aromatic amino acids, including phenylalanine, tyrosine, and tryptophan, are metabolized in the body, they generate ketones or biogenic amines that can stimulate insulin secretion and lower blood glucose levels [47]. Additionally, specific amino acids such as arginine, serine, and lysine, which are commonly found in proteins, can improve insulin sensitivity and promote glucose uptake through different metabolic pathways after ingestion, thereby affecting blood sugar levels [48]. Glycine can also promote insulin secretion, thereby reducing blood glucose levels and decreasing the peak value and AUC of postprandial blood glucose [49,50]. We employed a multiple regression analysis to analyze the relationship between the IAUC of blood glucose from 0 to 120 min after eating and various nutritional components. The results demonstrated that the content of lysine was negatively correlated with IAUC in gluten-containing and GF rice cakes with added 15% plant protein (p = 0.013 < 0.05). Glycine was significantly negatively correlated with IAUC in GF rice cakes with added 5%, 10%, and 15% pea protein and pure rice flour GF rice cakes (p = 0.003 < 0.01). In the future, we will further study the different effects of the intake of lysine and glycine on blood sugar levels. Apart from the types and amounts of amino acids, other nutritional components in food, such as fat, and an individual’s metabolic status can also affect postprandial blood glucose levels. An increase in fat content in food leads to a slower rise in blood glucose levels, and the peak value of postprandial blood glucose is also lowered. This suggests that the AUC of postprandial blood glucose is accordingly reduced. We found that the fat content in GF rice cakes with added 5%, 10%, and 15% soy protein and pure rice flour GF rice cakes was significantly negatively correlated with IAUC (p = 0.002 < 0.01), and an increase in fat content in food led to a significant decrease in IAUC at 2 h after the meal.

In this study, we used captive nonhuman primates, specifically the cynomolgus monkeys, as experimental animals to investigate the effects of gluten-containing and GF foods on postprandial blood glucose response. These nonhuman primates share many biological similarities with humans and exhibit similar morphology and functionality, making them a crucial animal model for such studies. Furthermore, their diet is relatively homogeneous, consisting mainly of various grains as well as supplemented nutrients such as milk, powdered milk, eggs, fish meal, bone meal, and salt. Disinfected vegetables and fruits are also added to ensure that the animals receive sufficient amounts of nutrients, such as vitamin C and minerals. We can ensure the relative stability of nutrient content in the feed through diversified feeding regimes. We also provide appropriate amounts of animal-sourced food to animals to ensure that they consume more than 50 types of nutrients, including carbohydrates, proteins, fats, vitamins, and minerals. We control the amount of food intake, especially for long-term captive monkeys, where overeating may lead to digestive problems or obesity. Nonhuman primates have a long history of application in translational medicine research, particularly in exploring the mechanisms underlying metabolic diseases and evaluating novel therapeutic approaches. Additionally, their longer lifespan facilitates the investigation and monitoring of the effects of long-term dietary patterns on animal health indices, such as gluten-containing and GF diets, high-sugar diets, high-fat diets, and so forth.

## 5. Conclusions

Among gluten-containing or GF foods made by mixing pea protein, rice protein, soy protein, wheat protein, and rice flour in varying ratios, GF rice cakes containing 15% pea protein exhibit a lower GI, lower postprandial 0 to 120 min IAUC, and potential for hypoglycemic effects. These findings can be applied to the development of GF foods using pea protein and rice flour as ingredients. Since GF products can be formulated with various alternative ingredients and further improved, more research is needed to determine the optimal substitutes for GF products to maintain blood glucose levels and promote health. Considering the increasing prevalence of CD, adhering to a lifelong GF diet remains the cornerstone of treating this disease. For patients with both T1DM and CD, not only a low-GI GF diet but also professional dietary counseling is necessary. Further research and development are needed in the GF product domain, especially for individuals with gluten sensitivity, diabetes, and neurodegenerative disease prevention needs.

## Figures and Tables

**Table 1:** Animals used in this study.

Animal Number	Sex	Age	Weight/kg	Sitting Height/cm	BMI	FBG (Fasting Blood Glucose)	Routine Stool Test
Healthy Animal-1	♂	16	9.71	53.0	34.6	2.95	Normal
Healthy Animal-2	♂	14	8.29	49.0	34.5	3.68	Normal
Healthy Animal-3	♂	15	8.44	51.0	32.4	2.93	Normal
Healthy Animal-4	♂	16	6.86	46.0	32.4	3.71	Normal
Healthy Animal-5	♂	14	7.14	47.0	32.3	3.77	Normal
Healthy Animal-6	♂	15	8.35	51.0	32.1	3.96	Normal
Healthy Animal-7	♂	15	7.68	49.0	32.0	3.30	Normal
Healthy Animal-8	♂	13	7.60	49.0	31.7	4.23	Normal
Healthy Animal-9	♂	16	6.87	47.0	31.1	3.84	Normal
Healthy Animal-10	♂	15	7.05	48.0	30.6	2.68	Normal
Healthy Animal-11	♂	12	6.94	48.0	30.1	3.53	Normal
Healthy Animal-12	♂	16	7.00	49.0	29.2	3.34	Normal

**Table 2:** Materials used in this study.

Materials/Instruments	Manufacture Factory
Thai fragrant rice	Siam Grains Co. (Bangkok, Thailand)
Wheat protein (WPI)	Your Health Store (London, UK)
Rice protein (RPI)	Xian Dongfeng Biotechnology Co., Ltd. (Xian, China)
Soy protein (SPI)	Linyi Shan Song Biological Products Co., Ltd. (Linyi, China)
Pea protein (PPI)	Roquette (China) Nutrition Food Co., Ltd. (Lianyungang, China)
Low-acyl gellan gum (Kelco, UK)	Guangdong Qian Heng Sheng Wu Technology Co., Ltd. (Guangzhou, China)
Sugar-free sweetener	Anhui Shu Jun Biotechnology Co., Ltd. (Hefei, China)
Food aromatics (peach flavor)	Hangzhou Bai Rui Flavor & Fragrance Co., Ltd. (Hangzhou, China)
Food-grade anhydrous glucose	Xiwang food co., Ltd. (Binzhou, China)
Blood glucose test paper and blood glucose meter	Roche Diagnostics in Germany (Basel, Switzerland)

**Table 3:** Test food formulation.

Rice Cake	Rice Cake Type	Rice Flour	Protein	Water
Control	Thai fragrant rice	35	–	65
5% PI	Thai fragrant rice + 5% PI	30	5	65
10% PI	Thai fragrant rice + 10% PI	25	10	65
15% PI	Thai fragrant rice + 15% PI	20	15	65

**Table 4:** Food feeding cycle.

Day	Diet Group
1	Control
2–4	Washout period
5	Thai fragrant rice + 15% wheat protein
6–8	Washout period
9	Thai fragrant rice + 10% wheat protein
10–12	Washout period
13	Thai fragrant rice + 5% wheat protein
14–16	Washout period

**Table 5:** Glycemic index (GI) of the 13 gluten-containing and GF tested foods (mean ± SE).

IDX	Food Type	GI (%)	Notes
1	Thai fragrant rice + 15% pea protein	29.16 ± 7.96	Gluten-free
2	Thai fragrant rice + 15% wheat protein	33.27 ± 17.19	Gluten
3	Thai fragrant rice + 10% soy protein	34.95 ± 21.62	Gluten-free
4	Thai fragrant rice + 10% rice protein	35.28 ± 12.40	Gluten-free
5	Thai fragrant rice + 10% wheat protein	35.60 ± 16.24	Gluten
6	Thai fragrant rice + 5% rice protein	37.24 ± 10.55	Gluten-free
7	Thai fragrant rice	37.46 ± 12.83	Gluten-free
8	Thai fragrant rice + 10% pea protein	37.76 ± 15.40	Gluten-free
9	Thai fragrant rice + 15% rice protein	37.86 ± 15.54	Gluten-free
10	Thai fragrant rice + 5% pea protein	38.49 ± 18.63	Gluten-free
11	Thai fragrant rice + 5% wheat protein	40.67 ± 15.36	Gluten
12	Thai fragrant rice + 5% soy protein	42.89 ± 15.29	Gluten-free
13	Thai fragrant rice + 15% soy protein	44.35 ± 17.68	Gluten-free

**Table 6:** Incremental area under the blood glucose curve (IAUC) for 13 gluten-containing and gluten-free tested foods 0–120 min postprandial (mean ± SE).

IDX	Food Type	0- to 120-min IAUC (mmol∙min/L)	Notes
1	Thai fragrant rice + 15% pea protein	499.81 ± 34.46	Gluten-free
2	Thai fragrant rice + 10% soy protein	516.69 ± 27.82	Gluten-free
3	Thai fragrant rice + 10% pea protein	519.25 ± 36.94	Gluten-free
4	Thai fragrant rice + 10% rice protein	521.63 ± 12.06	Gluten-free
5	Thai fragrant rice + 15% rice protein	530.50 ± 14.65	Gluten-free
6	Thai fragrant rice + 15% wheat protein	533.19 ± 34.89	Gluten
7	Thai fragrant rice	534.91 ± 22.51	Gluten-free
8	Thai fragrant rice + 5% rice protein	536.88 ± 16.07	Gluten-free
9	Thai fragrant rice + 10% wheat protein	540.00 ± 31.83	Gluten
10	Thai fragrant rice + 5% pea protein	542.19 ± 38.78	Gluten-free
11	Thai fragrant rice + 5% wheat protein	549.50 ± 48.80	Gluten
12	Thai fragrant rice + 5% soy protein	557.31 ± 12.94	Gluten-free
13	Thai fragrant rice + 15% soy protein	572.94 ± 72.74	Gluten-free

**Table 7:** Protein, starch, fat, and ash contents of 13 gluten-containing and gluten-free tested foods.

Name of the Food	%				
	Moisture Content	Protein	Starch	Fat	Ash Content
Thai fragrant rice + 15% pea protein	58.46	7.01	26.87	1.89	0.0062
Thai fragrant rice + 15% wheat protein	62.15	7.94	26.13	1.59	0.0021
Thai fragrant rice + 10% soy protein	60.2	6.21	27.27	2.23	0.056
Thai fragrant rice + 10% rice protein	53.29	7.07	32.84	0.08	0.0037
Thai fragrant rice + 10% wheat protein	61.5	5.73	25.98	0.16	0.0032
Thai fragrant rice + 5% rice protein	60.79	6.1	29.02	0.019	0.057
Thai fragrant rice	60.42	4.34	30.31	1.41	0.0071
Thai fragrant rice + 10% pea protein	63.41	7.03	21.18	2.39	0.18
Thai fragrant rice + 15% rice protein	59.49	7.48	25.88	0.52	0.051
Thai fragrant rice + 5% pea protein	69.2	5.81	28.86	2.23	0.073
Thai fragrant rice + 5% wheat protein	61.15	5.26	27.19	2.12	0.034
Thai fragrant rice + 5% soy protein	54.38	4.35	35.77	0.016	0.0197
Thai fragrant rice + 15% soy protein	57.32	5.49	32.42	0.49	0.12

**Table 8:** Levels of all amino acids in the 13 gluten-containing and gluten-free tested foods.

Name of the Food	mg/kg															
	Aspartic Acid	Glutamic Acid	Serine	Histidine	Glycine	Threonine	Arginine	Alanine	Tyrosine	Valine	Methionine	Phenylalanine	Isoleucine	Leucine	Lysine	Proline
Thai fragrant rice + 15% pea protein	5310.4	7463.11	2009.7	1266.25	585.76	1480.77	4959.79	2848.66	2601.35	4299.89	655.89	3131.28	3731.55	6227.55	2004.9	2835.16
Thai fragrant rice + 15% wheat protein	2239.2	11,079.03	1682.77	933.98	402.06	1002.81	2933.35	2020.12	2119.02	3200.9	684.45	2551.48	2784.38	4778.49	1154.88	5148.36
Thai fragrant rice + 10% soy protein	2665.79	4232.14	1207.7	910.81	380.64	856.42	2973.75	1788.16	1686.06	2550.4	674.14	1731.1	2128.94	3604.32	1286.41	1882.02
Thai fragrant rice + 10% rice protein	4132.29	7413.59	1885.49	1156.76	581.51	1329.19	4790.29	3038.97	2920.99	4412.08	892.94	2944.69	3272.1	5901.15	1446.18	2697.84
Thai fragrant rice + 10% wheat protein	2125.75	8874.5	1487.7	832.72	362.13	888.66	2865.46	2021.44	1957.96	3003.53	671.31	2179.51	2552.43	4361.47	1133.44	4109.98
Thai fragrant rice + 5% rice protein	2523.87	4704.83	1223.83	861.03	328.99	782.63	3252.5	2132.78	2030.54	3011.59	636.69	1844.42	2262.67	3869.58	1180.44	1884.41
Thai fragrant rice	1381.39	2569.24	745.58	550.75	259.73	454.53	2000.36	1298.79	1178.58	1859.16	431.59	1049.68	1449.13	2294.62	1002.23	1452.19
Thai fragrant rice + 10% pea protein	3907.92	5903.82	1622.68	991.99	445.54	1141.04	3970.39	2303.41	2115.37	3397.79	573.36	2305.62	2954.87	4864.49	1673.19	2259.08
Thai fragrant rice + 15% rice protein	5020.32	8704.48	2239.32	1316.82	646.68	1567.82	5559.87	3552.88	3507.31	5138.93	917.05	3513.5	3857.78	6980.23	1537.56	3010.23
Thai fragrant rice + 5% pea protein	2807.26	4179.93	1284.74	881.82	334.82	893.94	3180.02	1903.59	1709.35	2789.17	555.32	1787.46	2288.68	3795.22	1306.29	1791.83
Thai fragrant rice + 5% wheat protein	1697.57	5375.74	1149.51	763.46	302.17	691.63	2327.97	1555.28	1482.01	2332.46	532.94	1547.26	1929.28	3167.4	1063.21	2687.88
Thai fragrant rice + 5% soy protein	2017.43	3493.02	1029.2	800	305.13	690.87	2536.78	1530.51	1381.38	2175.67	475.59	1355.36	1811.25	2904.04	1106.46	1426.73
Thai fragrant rice + 15% soy protein	2892.74	4977.32	1201.52	846.13	338.22	778.69	3129.62	1983.19	1707.99	2790.74	560.72	1846.37	2396.84	3865.46	1326.54	2102.3

## Data Availability

The data presented in this study are available within the article. The raw data are available on request from the corresponding author.
